# Review: predatory soil mites as biocontrol agents of above- and below-ground plant pests

**DOI:** 10.1007/s10493-022-00723-w

**Published:** 2022-08-08

**Authors:** Giuditta M. Beretta, Jacques A. Deere, Gerben J. Messelink, Karen Muñoz-Cárdenas, Arne Janssen

**Affiliations:** 1 Department of Evolutionary and Population Biology, Institute for Biodiversity and Ecosystem Dynamics, University of Amsterdam, Amsterdam, The Netherlands; 2 Business Unit Greenhouse Horticulture, Wageningen University and Research, Bleiswijk, The Netherlands; 3 Department of Entomology, Federal University of Viçosa, Viçosa, Brazil

**Keywords:** Biological control, Alternative prey, Litter, Mesostigmata, Phytoseiidae, Alternative food

## Abstract

Biological pest control is becoming increasingly important for sustainable agriculture. Although many species of natural enemies are already being used commercially, efficient biological control of various pests is still lacking, and there is a need for more biocontrol agents. In this review, we focus on predatory soil mites, their role as natural enemies, and their biocontrol potential, mainly in vegetable and ornamental crops, with an emphasis on greenhouse systems. These predators are still underrepresented in biological control, but have several advantages compared to predators living on above-ground plant parts. For example, predatory soil mites are often easy and affordable to mass rear, as most of them are generalist predators, which also means that they may be used against various pests and can survive periods of pest scarcity by feeding on alternative prey or food. Many of them can also endure unfavourable conditions, making it easier for them to establish in various crops. Based on the current literature, we show that they have potential to control a variety of pests, both in greenhouses and in the field. However, more research is needed to fully understand and appreciate their potential as biocontrol agents. We review and discuss several methods to increase their efficiency, such as supplying them with alternative food and changing soil/litter structure to enable persistence of their populations. We conclude that predatory soil mites deserve more attention in future studies to increase their application in agricultural crops.

## Introduction

The control of pests in vegetable and ornamental crops is still a topic of major concern worldwide. For this, the use of biological control is an increasingly common practice, thus reducing the use of chemical pesticides (Ehlers 1996; van Lenteren 2012). This change is partly caused by the development of pesticide resistance by certain pests, partly due to consumer demands for pesticide-free products, and partly a consequence of the introduction of bumble bees for pollination in greenhouse crops such as tomato, which is incompatible with the use of chemical pesticides (Ehlers 1996; Matson et al. 1997; Isman 2006; Velthuis and van Doorn 2006; van Lenteren 2012). Many natural enemies of pests have been studied with respect to their suitability for biological control, and a number of them are used in a variety of crops. Despite the increased numbers of natural enemies commercially available for biological control, there is still a need for new candidates, partly because of the emergence of new pests, but also because the current natural enemies are not sufficiently efficient in all crops and under all conditions (Ehlers 1996; van Lenteren 2012). Predatory mites are among the most frequently used biocontrol agents of thrips, whiteflies and pest mites and are increasingly used for other pests (McMurtry and Croft 1997; Nomikou et al. 2002; Gerson and Weintraub 2007; Pijnakker and Ramakers 2009; McMurtry et al. 2013; Moreira and de Moraes 2015). In particular, phytoseiid mites have proven to be efficient natural enemies (Huffaker and Kennett 1953; Bravenboer and Dosse 1962; Ramakers 1980; van Rijn et al. 1999; Lesna et al. 2000; Nomikou et al. 2002, 2010; Messelink et al. 2008; McMurtry et al. 2013; Hoogerbrugge et al. 2014; Leman and Messelink 2015; Muñoz-Cárdenas 2017). However, this success is due to only a few predatory mite species compared to the large number of species described and an undoubtedly large reservoir of undescribed species. Hence, there is still a vast unexplored potential in this group. In particular, predatory soil mites are potentially highly effective biocontrol agents but have received less attention than their above-ground counterparts (Gillespie and Quiring 1990; Chambers et al. 1993; Wright and Chambers 1994; Lesna et al. 1996, 2000; Berndt et al. 2004a, b; Castilho et al. 2009; Moreira et al. 2015; Muñoz-Cárdenas 2017).

Soil mites are a very heterogeneous group, including prey, scavengers and predators. The latter are usually polyphagous; they can feed on many different pests and are therefore potentially versatile natural enemies (Berndt et al. 2004b; Messelink and de Kogel 2005; Gerson and Weintraub 2007; Messelink and van Holstein-Saj 2008; de Moraes et al. 2015). For example, the laelapid predators *Hypoaspis* (*Geolaelaps*) *aculeifer* (Canestrini) (for full taxonomic details see Table 1) and *Stratiolaelaps* (= *Hypoaspis*) *scimitus* (*miles*) (Womersley), as well as *Macrocheles robustulus* (Berlese) of the Macrochelidae family are used to control various species of edaphic pests (Gerson and Weintraub 2007; Messelink and Holstein-Saj 2008). Predatory soil mites are not only used to control pests inhabiting the soil, but also above-ground plants pests with so-called edaphic stages that inhabit the soil, for example the pupal stage of Western flower thrips, *Frankliniella occidentalis* (Pergande) (Berndt et al. 2004b; Wiethoff et al. 2004; Messelink and de Kogel 2005; Messelink and van Holstein-Saj 2008). Indeed, many above-ground plant pests do have life stages that occur in the soil (Gullino and Wordlow 1990), so predatory soil mites may control these pests.

**Table 1:** Predatory soil mites and the pests they can (potentially) control

Predators	Pests
*Hypoaspis* (*Geolaelaps*) *aculeifer* (Canestrini) (Parasitiformes: Mesostigmata: Lealapidae)	*Frankliniella occidentalis* (Pergande) (Western flower thrips) (Condylognatha: Thysanoptera: Thripidae)^6,7,14,15,29^ *Thrips tabaci* Lindeman (onion thrips) (Condylognatha: Thysanoptera: Thripidae)^9^ *Pezothrips kellyanus* (Bagnall) (citrus thrips) (Condylognatha: Thysanoptera: Thripidae)^30^ Sciarid flies and other Diptera^1,14,17^ *Rhizoglyphus robini* Claparède (Acariformes: Sarcoptiformes: Acaridae)^19,20^ *Tyrophagus similis V*olgin (bulb mite) (Acariformes: Sarcoptiformes: Acaridae)^18^ Springtails^4,16^ *Duponcheria fovealis* (Zeller) (southern European marshland pyralid) (Amphiesmenoptera: Lepidoptera: Crambidae)^22^
*Stratiolaelaps* (*Hypoaspis*) *scimitus* (*miles*) (Womersley) (Parasitiformes: Mesostigmata: Lealapidae)	*Frankliniella occidentalis*^6,7,15,28,32,35,36,40^ *Thrips tabaci* ^35^ Sciarid flies^8,10,13,34,38^ *Delia radicum* L. (cabbage root fly) (Panorpida: Diptera: Anthomyiidae) ^23^ *Duponcheria fovealis*^23^ *Meloidogyne incognita* (Kofoid & White) (root-knot nematode) (Rhabditica: Panagrolaimida: Meloidogynidae) ^3,39^
*Neoseiulus barkeri* Hughes (Parasitiformes: Mesostigmata: Phytoseiidae)	*Frankliniella occidentalis*^33^ *Thrips tabaci*^36^ *Steneotarsonemus laticeps* (Halbert) (Acariformes: Trombidiformes: Tarsoneminae)^4,22^
*Neoseiulus paspalivorus* (De Leon) (Parasitiformes: Mesostigmata: Phytoseiidae)	*Aceria tulipae* (Keifer) (dry bulb mite) (Acariformes: Trombidiformes: Eriophyidae)^21,33^
*Macrocheles robustulus* (Berlese) (Parasitiformes: Mesostigmata: Macrochelidae)	*Frankliniella occidentalis*^25,31^
*Cosmolaelaps jaboticabalensis* Moreira, Klompen & Moraes (Parasitiformes: Mesostigmata: Laelapidae)	*Frankliniella occidentalis*^26^
*Cosmolaelaps* *sabelis* (Sierra-Monroy et al. 2021; in press) (Parasitiformes: Mesostigmata: Laelapidae)	*Frankliniella occidentalis*^27^
*Balaustium leanderi* (Haitlinger, 2000) comb.nov. (Actinotrichida: Trombidifromes: Erythraeidae)	*Frankliniella occidentalis*^27^
*Blattisocius dolichus* Ma (Parasitiformes: Mesotigmata: Blattisociidae)	*Radophulus similis* (Cobb) Thorne (burrowing nematode) (Rhabditica: Panagrolaimida: *Pratylenchidae*)^11^ *Meloidogyne incognita*^37^
*Geolaelaps gillespiei* n. sp. (Parasitiformes: Mesostigmata: Lealapidae)	*Frankliniella occidentalis*^5^
*Parasitus bitoberosus* Karg (Parasitiformes: Mesotigmata: Parasitidae)	*Thrips tabaci*^9^
*Lasioseius fimetorum* Karg (Parasitiformes: Mesostigmata: Ascidae)	*Frankliniella occidentalis*^12^
*Cunaxa capreolus* (Berlese) (Acariformes: Trombidiformes: Cunaxidae)	*Meloidogyne incognita*^2^ *Tylenchulus semipenetrans* Cobb (citrus nematode) (Tylenchina: Tylenchida: Tylenchulidae)^2^

Table reference list: 1 Ajvad et al. (2018); 2 Al-Azzazy and Al-Rehiayani (2022); 3 Azevedo et al. (2020); 4 Baatrup et al. (2006); 5 Beaulieu (2009); 6 Berndt et al. (2004a); 7 Berndt et al. (2004b); 8 Castilho et al. (2009); 9 Castro-López and Martínez-Osorio (2021); 10 Chambers et al. (1993); 11 Chen et al. (2013); 12 Enkegaard and Brødsgaard (2000); 13 Freire et al. (2007); 14 Gillespie and Quiring (1990); 15 Glockemann (1992); 16 Jensen et al. (2019); 17 Jess and Bingham (2004); 18 Kasuga et al. (2006); 19 Lesna et al. (1996); 20 Lesna et al. (2000); 21 Lesna et al. (2014); 22 Messelink and van Wensveen (2003); 23 Messelink and van Slooten (2004); 24 Messelink and van Holstein-Saj (2006); 25 Messelink et al. (2008); 26 Moreira et al. (2015); 27 Muñoz-Cárdenas et al. (2014); 28 Muñoz-Cárdenas (2017); 29 Navarro-Campos et al. (2016); 30 Navarro-Campos et al. (2020); 31 Pozzebon et al. (2015); 32 Rahman et al. (2012); 33 Sabelis et al. (2008); 34 Wright and Chambers (1994); 35 Wu et al. (2014); 36 Wu et al. (2016); 37 Xu et al. (2014); 38 Yan et al. (2022); 39 Yang et al. (2020); 40 Zhang et al. (2021)

Whereas predatory soil mites have received little attention, even fewer studies have investigated the ability of generalist predators to prey on both below- and above-ground pests. The below-ground food web may provide alternative food to these predators (Scheu 2001; von Berg et al. 2009; Muñoz-Cárdenas et al. 2014; Neher and Barbercheck 2019), which may result in the persistence of predator populations when above-ground prey and pests are scarce (Muñoz-Cárdenas 2017). Therefore, a system with predators connecting the below- and above-ground food webs can be advantageous for biological control (Scheu 2001; von Berg et al. 2009; Muñoz-Cárdenas 2017; Neher and Barbercheck 2019).

The aim of this paper is to give an overview of the use of predatory soil mites as biocontrol agents, their advantages and the practical problems encountered thus far when using them. We mainly focus on vegetable and ornamental crops in greenhouse systems, but we also discuss examples of field studies. We start with an overview of the strengths and challenges of biological control with predatory soil mites. Then follows a description of the main pests that are targeted with predatory soil mites and case studies in which such predators have been used. Lastly, we go through some key points for the improvement of biological control with predatory soil mites.

## Predatory soil mites as biocontrol agents

The efficiency of biocontrol agents depends on many aspects, such as the presence of alternative prey or food, abiotic conditions, the cropping system, and possible interactions with other predators (Glockemann 1992; van Schelt et al. 2002; Berndt et al. 2004a; Wiethoff et al. 2004; Buitenhuis and Shipp 2008; Pijnakker and Ramakers 2008; Hoogerbrugge et al. 2014; Messelink 2014; Hewitt et al. 2015; Saito and Brownbridge 2016; Pijnakker et al. 2017). The presence of alternative prey or food can have both positive and negative effects on biological control. Supplying alternative food may result in increased predator densities and better pest control (van Rijn et al. 2002). In other cases, however, the effects of alternative prey or food are still not clear (Berndt et al. 2004a). Other elements that might affect the efficacy of biocontrol systems are abiotic factors; it is important to consider that seasonal changes can affect pests and predators even in greenhouse systems (Steiner et al. 2011; Hewitt et al. 2015). The limited use of predatory soil mites as biocontrol agents is undoubtedly also caused by the fact that soil is often absent in modern greenhouse systems, which mainly use artificial substrates. This does not prevent the presence of pests, but will hinder the introduction of soil predators (Fransen 1992; Paulitz 1997; van Schelt and Mulder 2000; Cloyd and Zaborski 2004). Furthermore, not all soil types are favourable for predatory soil mites. Sandy soils, for example, can be too compact for predatory mites to move freely and localize prey (Lesna et al. 2000; Sabelis et al. 2008). Lastly, when considering the release of multiple predator species at once, it is important to account for the possible interactions between them (Rosenheim et al. 1995; Mills 2006). A possible scenario is that one of the two predators could actively prey on the second one as well as on the pest (Rosenheim et al. 1995; Janssen et al. 2006; Montserrat et al. 2008, 2012; Momen and Abdel-Khalek 2009; Choh et al. 2014), and this hyperpredation or intraguild predation may affect biological control (Vance-Chalcraft et al. 2007; but see Janssen et al. 2006). Alternatively, the two predators could compete for the pest and this could make two predators less efficient than one predator alone (Janssen et al. 1998; Wiethoff et al. 2004; Mills 2006).

Many predatory mites are generalists, are easy and affordable to mass rear, may be used against various pests and can survive periods of pest scarcity by feeding on alternative prey or food (Chambers et al. 1993; Wright and Chambers 1994; Lesna et al. 1996). Predatory soil mites are very resilient; they too can survive periods of low prey densities and they are adapted to various environmental conditions, making it easier for them to establish in various crops (Chambers et al. 1993; Wright and Chambers 1994; Berndt et al. 2004a; Wiethoff et al. 2004; Moreira et al. 2015). Furthermore, the presence of predators inhabiting the above-ground plant parts is not tolerated in the marketable product of greenhouse floriculture, and given that soil predators will not be present on the above-ground parts of cut flowers, they potentially become a viable option (Fransen 1992; Beerling 2008; Pijnakker and Ramakers 2008; Muñoz-Cárdenas 2017).

An additional element restricting the use of predatory soil mites is that research on these natural enemies is challenging; the methods to quantify densities of these mites give highly variable results (Sabu et al. 2011; Owens and Carlton 2015; Knapp et al. 2018). For example, the numbers of soil mites extracted with the frequently-used Berlese-Tullgren funnels are affected by the extraction period, which often seems to be chosen arbitrarily (Owens and Carlton 2015). Moreover, Knapp et al. (2018) observed that predatory soil mites may escape from the funnels and are sometimes capable of reproducing during the extraction process, making a proper estimate of their densities impossible. It is therefore essential to have standardized methodology with respect to extraction time and quantity and quality of the soil samples.

## Predator-prey systems

Biocontrol with predatory soil mites has been investigated for several pests including thrips and flies, which we review in this section. Many of the studies were done in laboratory settings, and not much is known on the effects of predatory soil mites under crop-growing conditions. Even though soil predators are present in a large number of greenhouses, information on their predation rates, life cycles, and their ability to reproduce on various pest diets is lacking, but crucial for improvement of biological control (Wright and Chambers 1994; Berndt et al. 2004a, b; Freire and de Moraes 2007; Messelink and van Holstein-Saj 2008; Moreira et al. 2015). Many pests, such as *F. occidentalis*, several mite species, springtails, sciarids and other flies, lepidopterans and nematodes, have at least one life stage in the soil/litter layer and are therefore difficult to control with above-ground predators (Gillespie and Quiring 1990; Glockemann 1992; Chambers et al. 1993; Wright and Chambers 1994; Lesna et al. 1996, 2000; Berndt et al. 2004a, b; Messelink and van Slooten 2004; Messelink and van Holstein-Saj 2006, 2008; Saito and Brownbridge 2016; Castro-López and Martínez-Osorio 2021), and are often resistant to pesticides. We first discuss some of these pests and then review the predatory soil mites that are candidate natural enemies for biocontrol of such pests.

Thrips are some of the most difficult pests to control, mainly due to their complex life cycles, cryptic behaviour, and their ability to counter-attack predators (Bakker and Sabelis 1989; Glockemann 1992; Sabelis and van Rijn 1997; Faraji et al. 2002; Janssen et al. 2002; Koschier and Sedy 2003; Berndt et al. 2004a, b; Magalhães et al. 2005; Thoeming and Poehling 2006; Boateng et al. 2014; Muñoz-Cárdenas et al. 2014; Wu et al. 2014, 2016; Pozzebon et al. 2015; Saito and Brownbridge 2016). Sciarid flies, lepidopteran pests, and plant parasitic nematodes attack both ornamental and vegetable crops (Gillespie and Quiring 1990; Chambers et al. 1993; Wright and Chambers 1994; Moens and Perry 2009; Stocks and Hodges 2012), whereas Delia flies are a problem in cabbage (Soroka et al. 2001) and freesia (G. Messelink, pers. obs.) and biological control of bulb mites is mainly focused on ornamentals, although vegetable crops such as garlic can also benefit from bulb mite control (Lesna et al. 1996, 2000; Díaz et al. 2000; Messelink and van Holstein-Saj 2006). Another group of pests that proved hard to control are springtails, which attack a wide variety of vegetable crops such as lettuce, broccoli, cauliflower and spinach, as well as winter grain crops and pastures (Roberts et al. 2011; Joseph et al. 2015). As with *F. occidentalis*, a characteristic of springtails is their ability to counter-attack the predators, hindering the establishment of effective biocontrol agents (Jensen et al. 2019).

### Candidates for biological control of thrips

*Thrips tabaci* Lindeman (onion thrips) and *F. occidentalis* are known to attack around 250 plant species, including cucumber, pepper, rose, chrysanthemum and many other vegetables and ornamentals (Glockemann 1992; Sabelis and van Rijn 1997; Messelink and de Kogel 2005; Wu et al. 2021). They damage plants by ovipositing in leaf tissue and by feeding on leaves and flowers (Koschier and Sedy 2003; Boateng et al. 2014). Moreover, they act as vectors of plant viruses (Tommasini and Maini 1995; Ullman et al. 2002; Brunner et al. 2004; Riley et al. 2011; Boateng et al. 2014; Muñoz-Cárdenas et al. 2014; Pozzebon et al. 2015). Several soil predatory mite species have been evaluated for their capacity to control the (pre)pupal stage of these and some other thrips species (see Table 1 for species names and their synonyms).

Laboratory experiments with *H. aculeifer* showed promising results for controlling *F. occidentalis*, especially compared with *S. scimitus* (Berndt et al. 2004a, b). Greenhouse experiments on cucumber, Saintpaulia, and Pelargonium confirmed the potential of these two laelapid predators, but with high release ratios (Gillespie and Quiring 1990; Glockemann 1992). *Hypoaspis aculeifer* also performed well as a biocontrol agent of *T. tabaci* on onion plants in greenhouse experiments (Castro-López and Martínez-Osorio 2021), and it was found to be a promising natural enemy for control of *Pezothrips kellyanus* (Bagnall) (citrus thrips) in the laboratory, greenhouses and in the field (Navarro-Campos et al. 2020). *Stratiolaelaps scimitus* successfully reduced the densities of *T. tabaci and F. occidentalis* on cucumber plants in greenhouse experiments (Wu et al. 2014), but these authors found it difficult to predict the efficacy of *S. scimitus*. *Macrocheles robustulus* was also found to effectively control *F. occidentalis* on ornamental plants and they were more effective than *H. aculeifer* (Messelink and van Holstein-Saj 2008). Another predatory soil mite that proved to perform better than *H. aculeifer* is *Parasitus bitoberosus* Karg, which successfully reduced *T. tabaci* densities by almost 80% in greenhouse experiments on onion plants when released at high densities (Castro-López and Martínez-Osorio 2021). *Neoseiulus barkeri* Hughes controlled *T. tabaci* in experiments on cucumber and sweet pepper plants in greenhouses (Ramakers 1980; de Klerk and Ramakers 1986; Wu et al. 2014). On cucumber, *N. barkeri* seemed to achieve better control of *T. tabaci* than *S. scimitus* (Wu et al. 2014). Although *N. barkeri* is known to also enter the soil (Messelink and van Holstein-Saj 2006; I. Lesna, pers. obs.), it is not clear whether it preyed on the edaphic stages of the thrips. Other potential predatory soil mites for control of *F. occidentalis* are *Balaustium leanderi* (Haitlinger, 2000), *Lasioseius fimetorum* Karg, and species of the genus *Cosmolaelaps* which are part of ongoing research (Enkegaard and Brødsgaard 2000; Muñoz-Cárdenas et al. 2014; Moreira et al. 2015; Muñoz-Cárdenas 2017).

### Candidates for biological control of sciarid flies (fungus gnats) and other Diptera

Sciarid flies attack plant roots and stems and are especially important pests in young ornamentals and vegetables with root systems that are not yet fully developed (Gillespie and Quiring 1990; Chambers et al. 1993; Wright and Chambers 1994). *Stratiolaelaps scimitus* and *H. aculeifer* are capable of controlling sciarid flies and other Diptera in ornamentals, cucumber plants and mushrooms (Gillespie and Quiring 1990; Chambers et al. 1993; Jess and Bingham 2004; Freire et al. 2007; Ajvad et al. 2018; Table 1). In addition, *S. scimitus* gave promising results when tested for its short-term effect of sciarid flies on Chinese chive plants (Yan et al. 2022). Better results might be obtained when combining soil solarization with a subsequent release of *S. scimitus* (Yan et al. 2022). In cyclamen, *M. robustulus* was more efficient in controlling sciarid flies than was *H. aculeifer* (Grosman et al. 2011). Lastly, Messelink and van Slooten (2004) documented that *S. scimitus* showed promising results in controlling larvae of the cabbage root fly, *Delia radicum* L., an important pest of root crops.

### Candidates for biological control of pest mites

Bulb mites, bulb scale mites and dry bulb mites are important pests of ornamental flower bulbs such as lilies, amaryllis and tulips. The main hurdle when targeting these pests is their ability to hide between the bulb scales, making it harder to be reached by predators (Lesna et al. 1996, 2000, 2014; Messelink and van Holstein-Saj 2006; Sabelis et al. 2007, 2008). *Hypoaspis aculeifer* is the primary biocontrol agent of bulb mites; the full list of predators against these pests is shown in Table 1. It was also shown to be effective against *Rhizoglyphus robini* Claparède on lily bulbs in the laboratory as well as in greenhouse and field experiments (Lesna et al. 1996, 2000; Table 1). *Neoseiulus barkeri* is a promising natural enemy of bulb scale mites, *Steneotarsonemus laticeps* (Halbert), in amaryllis bulbs (Messelink and van Holstein-Saj 2006; Table 1). This predatory mite can colonize both the above-ground plant parts and the soil underneath, showing its plasticity and ability to link both habitats (Messelink and van Holstein-Saj 2006). Lastly, there is the possibility of using predatory soil mites to control mite pests in crops other than bulbs. Not much information can be found on this topic, but encouraging results of *H. aculeifer* controlling *Tyrophagus similis* Volgin in spinach (Kasuga et al. 2006) show that there are possibilities of further using these predators.

### Candidates for biological control of springtails

Springtails (Collembola) are a major component of the soil fauna and are involved in the decomposition of organic matter and nutrient cycling (Baatrup et al. 2006), but they can also be pests in crops such as lettuce, beetroots and maize, as well as in pastures (Bishop et al. 2001; Roberts et al. 2011; Joseph et al. 2015; Joseph 2017; Jensen et al. 2019). In particular *H. aculeifer* demonstrated capacity to control springtails in the laboratory (Baatrup et al. 2006; Jensen et al. 2019; Table 1), but there are no studies on its effectiveness at larger spatial scales. However, the efficiency of predatory mites in controlling springtails can be reduced by the counterattack behaviour of certain springtail species (Jensen et al. 2019).

### Candidates for biological control of Lepidoptera

Lepidopteran pests with at least one edaphic life stage can be targets for control with predatory soil mites; a prime example is the European pepper moth, *Duponchelia fovealis* (Zeller). Its five larval stages reside preferably in the soil, but they can also be found on plant parts close to the soil (Blok and Messelink 2007; Stocks and Hodges 2012). They feed on plant stems, the lower plant leaves, and occasionally on roots (Blok and Messelink 2007; Stocks and Hodges 2012). Until now, *S. scimitus* and *H. aculeifer* have been studied for control of this pest (Messelink and van Wensveen 2003; Blok and Messelink 2007; Table 1). Both mites managed to control *D. fovealis*, but the predation rate of *S. scimitus* was slightly higher than that of *H. aculeifer*, probably due to the fact that the former mite prefers the top soil layer where most of the lepidopteran eggs and larvae are found (Messelink and van Wensveen 2003).

### Candidates for biological control of nematodes

Plant parasitic nematodes are pests in many agricultural crops; they affect plant growth and fruit production by attacking plant roots, causing lesions, cysts, and gall formation (Moens et al. 2009; Perry and Moens 2011; Sikora et al. 2018). In laboratory experiments, all mobile stages of the predatory soil mite *Cunaxa capreolus* (Berlese) successfully preyed on egg masses and second-instar larvae of the root knot nematode *Meloidogyne incognita* (Kofoid & White) and the citrus nematode *Tylenchulus semipenetrans* Cobb (Al-Azzazy and Al-Rehiayani 2022). The predatory soil mite *Blattisocius dolichus* Ma was able to complete its life cycle on a diet of the nematode *Radophulus similis* (Cobb) Thorne and control it in potted *Anthurium andreanum* plants (Chen et al. 2013; Table 1). It also showed good control of the root knot nematode *M. incognita* in pots with water spinach plants (*Ipomoea aquatica*, Xu et al. 2014; Table 1). *Stratiolaelaps scimitus* also controlled *M. incognita* in potted spinach plants (Yang et al. 2020; Table 1) and in tomato plants with free-living nematodes (*Rhabditella axei*) as alternative food (Azevedo et al. 2020; Table 1).

### Candidates for biological control of Coleoptera

Among Coleoptera pests, the Western corn root worm, *Diabrotica virgifera virgifera* LeConte (Endopterygota: Coleoptera: Chrysomelidae), is considered one of the major threats to agriculture. It feeds mainly on maize plants: the first instar damages the finer root hairs and the older instars damage the bigger nodal roots, whereas adults might also affect the reproductive plant organs (Branson et al. 1980; Spencer et al. 2009). This creates direct damage leading to yield loss (Riedell 1990; Sutter et al. 1990; Spike and Tollefson 1991; Godfrey et al. 1993; Gray et al. 2009). Laboratory studies showed promising results when testing predatory soil mites against this pest; field experiments, however, gave mixed results (Prischmann-Voldseth et al. 2011; Prischmann-Voldseth and Dashiell 2013; Pasquier et al. 2021a, b).

## How to improve biological control with predatory soil mites

### Biodiversity of the soil ecosystem

It is well known that increased biodiversity can promote biological pest control (Gurr et al. 2003; Bianchi et al. 2006; Scherber et al. 2010; Chaplin-Kramer et al. 2011; Woltz et al. 2012). Simplified landscapes with low biodiversity such as modern agricultural fields are often not varied enough to guarantee the presence and the successful action of natural enemies (Gurr et al. 2003; Bianchi et al. 2006; Chaplin-Kramer et al. 2011) and this holds for below-ground as well as above-ground biodiversity. The soil community is known to alter soil composition and nutrient availability, thus directly affecting plant quality and, indirectly, the performance and composition of the above-ground fauna (Scheu 2001; Neher and Barbercheck 2019). The establishment of links between below- and above-ground systems could lead to new solutions for biocontrol and a more unified understanding of the ecosystem associated with plants. Even without much knowledge of such links, there is general agreement that soil biodiversity is important, both in open fields and greenhouses (Gurr et al. 2003). There are many ways in which more varied habitats can be stimulated, for example with increased plant diversity in field margins and with cover crops (Gurr et al. 2003; Bianchi et al. 2006; Woltz et al. 2012).

### Soil structure

Changes in soil structure can affect natural enemies in the soil: the addition of a mulch layer to strawberry plants resulted in increased predatory soil mite densities through offering them protection against high temperatures and low humidity (Esteca et al. 2018, 2020). Consequently, this resulted in a decrease of spider mite densities. The litter layer is usually removed in many greenhouse crops because it can be a source of pests, pathogens, and toxic compounds (Mercier and Manker 2005; Cartenì et al. 2016). However, it could be kept to promote the establishment of predatory soil mite populations (Muñoz-Cárdenas et al. 2017; Walter and Stirling 2018; Esteca et al. 2020; Navarro-Campos et al. 2020). We suggest that applying litter or mulch to artificial substrates in soilless crops can promote the presence and persistence of soil predators, thus increasing their role in pest control. Lastly, highly degradable substrates could promote the soil microfauna, thus sustaining predator populations and resulting in more successful biological control (Blok and Messelink 2007).

### Adding alternative food to the soil

The addition of alternative food, such as pollen, *Artemia* (brine shrimp) cysts and moth eggs, to above-ground plant parts is becoming a common practice in many crops (Arijs and De Clercq 2001; Nomikou et al. 2002; van Rijn et al. 2002; Maoz et al. 2011; Delisle et al. 2015; Janssen and Sabelis 2015; Leman and Messelink 2015; Pijnakker et al. 2016; Ghasemzadeh et al. 2017; Warburg et al. 2019). Adding or maintaining alternative food or prey in the soil, the litter layer, or the mulch is likewise beneficial for soil predators (Elkins and Whitford 1982; Messelink and van Holstein-Saj 2008; von Berg et al. 2009; Muñoz-Cárdenas 2017; Esteca et al. 2018, 2020; Rueda-Ramírez et al. 2018, 2019; Neher and Barbercheck 2019; Azevedo et al. 2020), and has been shown to result in enhanced pest control (Muñoz-Cárdenas 2017; Muñoz-Cárdenas et al. 2017; Esteca et al. 2018, 2020; Rueda-Ramírez et al. 2018; Azevedo et al. 2020). Introducing alternative prey to the litter or mulch layer can promote the growth and establishment of predators in the absence of pests. The positive effect of a litter/mulch layer can also work in field crops, and the addition of alternative prey to these substrates could boost predator populations even more (Muñoz-Cárdenas 2017; Navarro-Campos et al. 2020).

The provisioning of alternative food results in an indirect interaction between the alternative food and the pest, driven by the actions of the shared predator. As such, this indirect interaction between the alternative food and pest can lead to lower predation on the pest, thus increasing pest densities (so-called apparent mutualism, Holt 1977), at least in the short term, because adding alternative food results in satiation of the predators. Indeed, several studies have shown the occurrence of apparent mutualism with a potential negative impact on biocontrol (Desneux and O’Neil 2008; van Maanen et al. 2012; Desneux et al. 2019). Consequently, this interaction may negate the positive impacts of alternative food provisioning. Given this, the quality and the frequency of provisioning alternative food would then play an important role in determining the extent of any beneficial effects of adding alternative food.

### Combining below-ground and above-ground predators and other control measures

The release of a combination of soil-dwelling natural enemies and predators occurring on the above-ground plant parts has repeatedly been suggested for more efficient pest control (Glockemann 1992; Wiethoff et al. 2004; Muñoz-Cárdenas et al. 2014). The idea is that the two predators would target different stages of pests with edaphic stages, resulting in more successful pest suppression. For example, Wiethoff et al. (2004) found that the soil mite *H. aculeifer* alone did not reduce *F. occidentalis* densities sufficiently, but control was more effective when combined with the plant-dwelling predatory mite *Amblyseius cucumeris* (Oudemans) (Mesostigmata: Phytoseiidae). On a similar note, it was found that successful control of *F. occidentalis* in cyclamen plants could be achieved by combining the predatory bug *Orius laevigatus* (Fieber) (Hemiptera: Anthocoridae) with either the predatory soil mite *M. robustulus*, or with entomoparasitic nematodes (Pozzebon et al. 2015). Similarly, *H. aculeifer* controlled *F. occidentalis* better together with entomopathogenic nematodes than alone (Premachandra et al. 2003a). These results are promising, but more studies have to be done to assess the possibility of negative interactions such as intraguild predation between the different natural enemies. In general, it is possible that intraguild predation between soil and above-ground predators hinders their effectiveness, but the possibilities for these predators to meet is likely lower than for predators that occur on the same plant parts (Northfield et al. 2017). Moreover, intraguild predation often does not seem to impede biological control above-ground (Janssen et al. 2006; Pochubay et al. 2015; but see Vance-Chalcraft et al. 2007). To date, there are few studies on intraguild predation between soil and leaf predators, possibly caused by soil food webs being understudied and partly because above- and below-ground food webs were often considered as two separate systems (Wardle et al. 2004).

It is also important to consider the possibility of combining biopesticides and microbial control agents with natural enemies (Waiganjo et al. 2011; Srinivasan 2012; Gonzalez et al. 2016; Saito and Brownbridge 2016; Soares et al. 2019). Waiganjo and colleagues (2011) found better control of aphids and diamondback moths, *Putella xylostella* (L.) (Lepidoptera: Plutellidae), with a combination of biopesticides and natural enemies. Results of such combinations are, however, often dependent on the type and concentration of the biopesticide. Rahman et al. (2012) showed successful control of *F. occidentalis* in strawberries when the biopesticide spinosad was applied 5–6 days prior to the release of leaf and soil predatory mites in various combinations. They observed no negative effects of spinosad on mite performance and development; however, the residual toxicity should be investigated further. Saito and Brownbridge (2016) found promising results at low concentrations of certain bioactive substances, but high concentrations were harmful for the predators and decreased their efficiency as biocontrol agents. Along the same line, control of the tomato leafminer *Tuta absoluta* (Meyrick) (Lepidoptera: Gelechiidae) with essential oils raised some concern on potential long-term effects of these products on natural enemies (Soares et al. 2019). A selling point for soil predators is that they may escape the side-effects of pesticides applied to above-ground plant parts (Beerling 2008; Pijnakker and Ramakers 2009). Even though the combination of chemicals and (soil) predators could be a good strategy, lethal and sublethal effects of pesticides affect predator-prey dynamics (Relyea and Edwards 2010; Cabral et al. 2011; Rasmussen et al. 2013) and will often not result in better pest control (Janssen and van Rijn 2021). In some cases, these chemical substances affect the fecundity and reproduction of the predator (Rasmussen et al. 2013). In other situations, pesticides slow down the mobility of pests to the advantage of a natural enemy (Cabral et al. 2011), but it is questionable whether this would also increase predation by soil predators. Ultimately, it is important to evaluate the compatibility of bioactive substances and predators on a case-by-case basis, testing for effects on the pest-natural enemy dynamics (Biondi et al. 2012, 2013; Saito and Brownbridge 2016).

Another approach is the combination of predatory soil mites and microbial agents to control pests. A positive example is the control of *F. occidentalis* in greenhouse experiments on eggplants when using granules of the entomopathogenic fungus *Beauveria bassiana* (Bals.-Criv.) Vuill. (Hypocreales: Cordycipitacecae) alone or in combination with *S. scimitus* and its alternative prey *Tyrophagus putrescentiae* (Schrank) (Astigmata: Acaridae) (Zhang et al. 2021). The combination treatment worked the best, however, there could be some competition between the predators due to the shared prey (thrips pupae). This aspect should be studied more to confirm the efficacy of such systems.

## Conclusions and perspectives

The use of predatory soil mites as biocontrol agents against edaphic prey is gaining attention; however, many more studies are needed for better understanding of the biology of these mites and their full potential as biocontrol agents. Moreover, more applied studies in greenhouses and outdoors are needed to assess the efficiency of these predators in commercial cropping systems. The current literature shows that predatory soil mites can be included in biocontrol programs, but methods must, and can be, improved. One such improvement is the use of a litter/mulching layer to increase the persistence of the predators in a cropping system (Muñoz-Cárdenas et al. 2017; Esteca et al. 2018, 2020; Walter and Stirling 2018; Navarro-Campos et al. 2020). Another is the supply of alternative food for the predators in the soil, guaranteeing persistence of their populations when pest densities are low. Several types of alternative food could be explored, for example, saprophytic nematodes seem a promising choice (Navarro-Campos et al. 2016). A further potentially successful strategy is the combination of soil and plant predators; however, their compatibility and predatory interactions need to be studied first (Glockemann 1992; Premachandra et al. 2003; Wiethoff et al. 2004). Likewise, an interesting avenue is to use soil predators to combat other soil pests such as root aphids (Wenninger 2011; Müller 2019). Furthermore, more research is needed on the effects of soil structure (Lesna et al. 2000; Jindo et al. 2020) and cropping systems on biocontrol (Chabert and Sarthou 2017). In conclusion, we argue that the use of predatory soil mites is a promising additional strategy for sustainable control of pests with soil-borne stages.

## References

[CR1] Ajvad FT, Madadi H, Michaud JP, Zafari D, Khanjani M (2018). Life table of *Gaeolaelaps aculeifer* (Acari: Laelapidae) feeding on larvae of *Lycoriella auripila* (Diptera: Sciaridae) with stage-specific estimates of consumption. Biocontrol Sci Technol.

[CR2] Al-Azzazy M, Al-Rehiayani S (2022). The soil mite *Cunaxa capreolus* (Acari: Cunaxidae) as a predator of the root-knot nematode, *Meloidogyne incognita* and the citrus Nematode, *Tylenchulus semipenetrans*: implications for biological control. Acarologia.

[CR3] Arijs Y, de Clercq P (2001). Rearing *Orius laevigatus* on cysts of the brine shrimp *Artemia franciscana*. Biol Control.

[CR4] Azevedo LH, Moreira MFP, Pereira GG (2020). Combined releases of soil predatory mites and provisioning of free-living nematodes for the biological control of root-knot nematodes on ‘Micro Tom tomato’. Biol Control.

[CR5] Baatrup E, Bayley M, Axelsen JA (2006). Predation of the mite *Hypoaspis aculeifer* on the springtail *Folsomia fimetaria* and the influence of sex, size, starvation, and poisoning. Entomol Exp Appl.

[CR6] Bakker FM, Sabelis MW (1989). How larvae of *Thrips tabaci* reduce the attack success of phytoseiid predators. Entomol Exp Appl.

[CR7] Beaulieu F (2009). Review of the mite genus Gaeolaelaps Evans & Till (Acari: Laelapidae), and description of a new species from North America, *G. gillespiei* n. sp. Zootaxa.

[CR8] Beerling E (2008). The switch to IPM in cut-chrysanthemum in the Netherlands. IOBC/WPRS Bull.

[CR9] Berndt O, Meyhöfer R, Poehling HM (2004). The edaphic phase in the ontogenesis of *Frankliniella occidentalis* and comparison of *Hypoaspis miles* and *Plypoaspis aculeifer* as predators of soil-dwelling thrips stages. Biol Control.

[CR10] Berndt O, Poehling HM, Meyhöfer R (2004). Predation capacity of two predatory laelapid mites on soil-dwelling thrips stages. Entomol Exp Appl.

[CR11] Bianchi FJJA, Booij CJH, Tscharntke T (2006). Sustainable pest regulation in agricultural landscapes: a review on landscape composition, biodiversity and natural pest control. Proc R Soc B Biol Sci.

[CR12] Biondi A, Desneux N, Siscaro G, Zappalà L (2012). Using organic-certified rather than synthetic pesticides may not be safer for biological control agents: selectivity and side effects of 14 pesticides on the predator *Orius laevigatus*. Chemosphere.

[CR13] Biondi A, Zappalà L, Stark JD, Desneux N (2013). Do biopesticides affect the demographic traits of a parasitoid wasp and its biocontrol services through sublethal effects?. PLoS ONE.

[CR14] Bishop AL, McKenzie HJ, Harris AM, Barchia IM (2001). Distribution and ecology of the lucerne flea, *Sminthurus viridis* (L.) (Collembola: Sminthuridae), in irrigated lucerne in the Hunter dairying region of New South Wales. Aust J Entomol.

[CR15] Blok C, Messelink GJ (2007). Improving control of *Duponchelia fovealis* (Lepidoptera: Pyralidae) by rooting media related strategies. Acta Hort.

[CR16] Boateng CO, Schwartz HF, Havey MJ, Otto K (2014). Evaluation of onion germplasm for resistance to iris yellow spot (Iris yellow spot virus) and onion thrips, *Thrips tabaci*. Southwest Entomol.

[CR17] Branson TF, Sutter GR, Fisher JR (1980). Plant response to stress induced by artificial infestations of western corn rootworm. Environ Entomol.

[CR18] Bravenboer L, Dosse G (1962). *Phytoseiulus riegeli* Dosse als Prädator Einiger Schadmilben aus der *Tetranychus urticae*-Gruppe. Entomol Exp Appl.

[CR19] Brunner PC, Chatzivassiliou EK, Katis NI, Frey JE (2004). Host-associated genetic differentiation in *Thrips tabaci* (Insecta; Thysanoptera), as determined from mtDNA sequence data. Heredity.

[CR20] Buitenhuis R, Shipp JL (2008). Influence of plant species and plant growth stage on *Frankliniella occidentalis* pupation behaviour in greenhouse ornamentals. J Appl Entomol.

[CR21] Cabral S, Soares AO, Garcia P (2011). Voracity of *Coccinella undecimpunctata*: effects of insecticides when foraging in a prey/plant system. J Pest Sci.

[CR22] Cartenì F, Bonanomi G, Giannino F (2016). Self-DNA inhibitory effects: underlying mechanisms and ecological implications. Plant Signal Behav.

[CR23] Castilho RC, de Moraes GJ, Silva ES (2009). The predatory mite *Stratiolaelaps scimitus* as a control agent of the fungus gnat *Bradysia matogrossensis* in commercial production of the mushroom *Agaricus bisporus*. Int J Pest Manag.

[CR24] Castro-López MA, Martínez-Osorio JW (2021). *Allium cepa* L. responses when *Gaeolaelaps aculeifer* Canestrini and *Parasitus bituberosus* Karg are used to control *Thrips tabaci* Lindeman. Revista Colombiana de Ciencias Hortícolas.

[CR25] Chabert A, Sarthou JP (2017). Practices of conservation agriculture prevail over cropping systems and landscape heterogeneity in understanding the ecosystem service of aphid biocontrol. Agric Ecosyst Environ.

[CR26] Chambers RJ, Wright EM, Lind RJ (1993). Biological control of glasshouse sciarid flies (*Bradysia* spp.) with the predatory mite, *Hypoaspis miles*, on cyclamen and poinsettia. Biocontrol Sci Technol.

[CR27] Chaplin-Kramer R, O’Rourke ME, Blitzer EJ, Kremen C (2011). A meta-analysis of crop pest and natural enemy response to landscape complexity. Ecol Lett.

[CR28] Chen YL, Xu CL, Xu XN (2013). Evaluation of predation abilities of *Blattisocius dolichus* (Acari: Blattisociidae) on a plant-parasitic nematode, *Radopholus similis* (Tylenchida: Pratylenchidae). Exp Appl Acarol.

[CR29] Choh Y, Takabayashi J, Sabelis MW, Janssen A (2014). Witnessing predation can affect strength of counterattack in phytoseiids with ontogenetic predator-prey role reversal. Anim Behav.

[CR30] Cloyd RA, Zaborski ER (2004). Fungus gnats, *Bradysia* spp. (Diptera: Sciaridae), and other arthropods in commercial bagged soilless growing media and rooted plant plugs. J Econ Entomol.

[CR31] de Klerk ML, Ramakers PMJ (1986). Monitoring population densities of the phytoseiid predator *Amblyseius cucumeris* and its prey after large scale introductions to control *Thrips tabaci* on sweet peppers. Mededelingen Faculteit Landbouwwetenschappen Rijksuniversiteit Gent.

[CR32] de Moraes GJ, Venancio R, dos Santos VL, Paschoal A, Carrillo D, de Moraes G, Peña J (2015). Potential of Ascidae, Blattisociidae and Melicharidae (Acari: Mesostigmata) as biological control agents of pest organisms. Prospects for biological control of plant feeding mites and other harmful organisms. Progress in Biological Control.

[CR33] Delisle JF, Brodeur J, Shipp L (2015). Evaluation of various types of supplemental food for two species of predatory mites, *Amblyseius swirskii* and *Neoseiulus cucumeris* (Acari: Phytoseiidae). Exp Appl Acarol.

[CR34] Desneux N, O’Neil RJ (2008). Potential of an alternative prey to disrupt predation of the generalist predator, *Orius insidiosus*, on the pest aphid, *Aphis glycines*, via short-term indirect interactions. Bull Entomol Res.

[CR35] Desneux N, Kaplan I, Yoo HJS (2019). Temporal synchrony mediates the outcome of indirect effects between prey via a shared predator. Entomol Generalis.

[CR36] Díaz A, Okabe K, Eckenrode CJ (2000). Biology, ecology, and management of the bulb mites of the genus *Rhizoglyphus* (Acari: Acaridae). Exp Appl Acarol.

[CR37] Ehlers RU (1996). Current and future use of nematodes in biocontrol: practice and commercial aspects with regard to regulatory policy issues. Biocontrol Sci Technol.

[CR38] Elkins NZ, Whitford WG (1982). The role of microarthropods and nematodes in decomposition in a semi-arid ecosystem. Oecologia.

[CR39] Enkegaard A, Brødsgaard HF (2000). *Lasioseius fimetorum*: a soil-dwelling predator of glasshouse pests?. BioControl.

[CR40] Esteca de CNF, Rodrigues LR, de Moraes GJ (2018). Mulching with coffee husk and pulp in strawberry affects edaphic predatory mite and spider mite densities. Exp Appl Acarol.

[CR41] Esteca de CNF, Trandem N, Klingen I (2020). Cereal straw mulching in strawberry—a facilitator of plant visits by edaphic predatory mites at night?. Diversity.

[CR42] Faraji F, Janssen A, Sabelis MW (2002). Oviposition patterns in a predatory mite reduce the risk of egg predation caused by prey. Ecol Entomol.

[CR43] Fransen JJ (1992). Development of integrated crop protection in glasshouse ornamentals. Pest Sci.

[CR44] Freire RAP, de Moraes GJ (2007). Description of a new species of *Cosmolaelaps berlese* (Acari: Laelapidae, Hypoaspidinae) from Brazil and its biological cycle. Int J Acarol.

[CR45] Freire RAP, de Moraes GJ, Silva ES (2007). Biological control of *Bradysia matogrossensis* (Diptera: Sciaridae) in mushroom cultivation with predatory mites. Exp Appl Acarol.

[CR46] Gerson U, Weintraub PG (2007). Mites for the control of pests in protected cultivation. Pest Manag Sci.

[CR47] Ghasemzadeh S, Leman A, Messelink GJ (2017). Biological control of *Echinothrips americanus* by phytoseiid predatory mites and the effect of pollen as supplemental food. Exp Appl Acarol.

[CR48] Gillespie DR, Quiring DM (1990). Biological control of fungus gnats, *Bradysia* spp. (Diptera: Sciaridae), and western flower thrips, *Frankliniella occidentals* (Pergande)(Thysanoptera: Thripidae), in greenhouses using a soil-dwelling predatory mite, *Geolaelaps* sp. nr. *aculeifer* (Canestrini). Can Entomol.

[CR49] Glockemann B (1992). Biological control of *Frankliniella occidentalis* on ornamental plants using predatory mites. EPPO Bull.

[CR50] Godfrey LD, Meinke LJ, Wright RJ (1993). Vegetative and reproductive biomass accumulation in field com: response to root injury by western com rootworm (Coleoptera: Chrysomelidae). J Econ Entomol.

[CR51] Gonzalez F, Tkaczuk C, Dinu MM (2016). New opportunities for the integration of microorganisms into biological pest control systems in greenhouse crops. J Pest Sci.

[CR52] Gray ME, Sappington TW, Miller NJ, Moeser J, Bohn MO (2009). Adaptation and invasiveness of western corn rootworm: intensifying research on a worsening pest. Ann Rev Entomol.

[CR53] Grosman AH, Messelink GJ, de Groot E (2011). Combined use of a mulch layer and the soil-dwelling predatory mite *Macrocheles robustulus* (Berlese) enhance the biological control of sciarids in potted plants. Biocontrol.

[CR54] Gullino ML, Wordlow L (1990). Ornamentals.

[CR55] Gurr GM, Wratten SD, Luna JM (2003). Multi-function agricultural biodiversity: pest management and other benefits. Basic Appl Ecol.

[CR56] Hewitt LC, Shipp L, Buitenhuis R, Scott-Dupree C (2015). Seasonal climatic variations influence the efficacy of predatory mites used for control of western flower thrips in greenhouse ornamental crops. Exp Appl Acarol.

[CR57] Holt D (1977). Predation, apparent competition, and the structure of prey communities. Theor Popul Biol.

[CR58] Hoogerbrugge H, Lenferink KO, van Houten Y, Bolckmans K (2014). Screening of three phytoseiid mite species as biocontrol agents of *Echinothrips americanus*. IOBC/WPRS Bull.

[CR59] Huffaker CB, Kennett CE (1953). Developments toward biological control of cyclamen mite on strawberries in California. J Econ Entomol.

[CR60] Isman MB (2006). Botanical insecticides, deterrents, and repellents in modern agriculture and an increasingly regulated world. Ann Rev Entomol.

[CR61] Janssen A, Sabelis MW (2015). Alternative food and biological control by generalist predatory mites: the case of *Amblyseius swirskii*. Exp Appl Acarol.

[CR62] Janssen A, van Rijn PCJ (2021). Pesticides do not significantly reduce arthropod pest densities in the presence of natural enemies. Ecol Lett.

[CR63] Janssen A, Pallini A, Venzon M, Sabelis MW (1998). Behaviour and indirect interactions in food webs of plant-inhabiting arthropods. Exp Appl Acarol.

[CR64] Janssen A, Faraji F, Van der Hammen T (2002). Interspecific infanticide deters predators. Ecol Lett.

[CR65] Janssen A, Montserrat M, HilleRisLambers R, Brodeur J, Boivin G (2006). Intraguild predation usually does not disrupt biological control. Trophic and guild interactions in biological control.

[CR66] Jensen K, Toft S, Sørensen JG (2019). Prey-specific experience affects prey preference and time to kill in the soil predatory mite *Gaeolaelaps aculeifer* Canestrini. Biol Control.

[CR67] Jess S, Bingham JFW (2004). Biological control of sciarid and phorid pests of mushroom with predatory mites from the genus *Hypoaspis* (Acari: Hypoaspidae) and the entomopathogenic nematode *Steinernema feltiae*. Bull Entomol Res.

[CR68] Jindo K, Olivares FL, da Paixao Malcher DJ (2020). From lab to field: role of humic substances under open-field and greenhouse conditions as biostimulant and biocontrol agent. Front Plant Sci.

[CR69] Joseph SV (2017). Influence of plant age, temperature, and moisture on *Protaphorura fimata* feeding injury on lettuce in the Salinas Valley of California, USA. Entomol Exp Appl.

[CR70] Joseph SV, Bettiga C, Ramirez C, Soto-Adames FN (2015). Evidence of *Protaphorura fimata* (Collembola: Poduromorpha: Onychiuridae) feeding on germinating lettuce in the salinas valley of California. J Econ Entomol.

[CR71] Kasuga S, Kanno H, Amano H (2006). Development, oviposition, and predation of *Hypoaspis aculeifer* (Acari: Laelapidae) feeding on *Tyrophagus similis* (Acari: Acaridae). J Acarological Soc Japan.

[CR72] Knapp M, van Houten Y, van Baal E, Groot T (2018). Use of predatory mites in commercial biocontrol: current status and future prospects. Acarologia.

[CR73] Koschier EH, Sedy KA (2003). Labiate essential oils affecting host selection and acceptance of *Thrips tabaci* Lindeman. Crop Prot.

[CR74] Leman A, Messelink GJ (2015). Supplemental food that supports both predator and pest: a risk for biological control?. Exp Appl Acarol.

[CR75] Lesna I, Sabelis MW, Conijn CGM (1996). Biological control of the bulb mite, *Rhizoglyphus robini*, by the predatory mite, *Hypoaspis aculeifer*, on lilies: predator-prey interactions at various spatial scales. J Appl Ecol.

[CR76] Lesna I, Conijn CGM, Sabelis MW, van Straalen NM (2000). Biological control of the bulb mite, *Rhizoglyphus robini*, by the predatory mite, *Hypoaspis aculeifer*, on lilies: predator-prey dynamics in the soil, under greenhouse and field conditions. Biocontrol Sci Technol.

[CR77] Lesna I, da Silva FR, Sato Y (2014). *Neoseiulus paspalivorus*, a predator from coconut, as a candidate for controlling dry bulb mites infesting stored tulip bulbs. Exp Appl Acarol.

[CR78] Magalhães S, Janssen A, Montserrat M, Sabelis MW (2005). Prey attack and predators defend: counterattacking prey trigger parental care in predators. Proc R Soc B Biol Sci.

[CR79] Maoz Y, Gal S, Argov Y (2011). Biocontrol of persea mite, *Oligonychus perseae*, with an exotic spider mite predator and an indigenous pollen feeder. Biol Control.

[CR80] Matson PA, Parton WJ, Power AG, Swift MJ (1997). Agricultural intensification and ecosystem properties. Science.

[CR81] McMurtry JA, Croft BA (1997). Life-styles of Phytoseiid mites and their roles in biological control. Ann Rev Entomol.

[CR82] McMurtry JA, De Moraes GJ, Sourassou NF (2013). Revision of the lifestyles of phytoseiid mites (Acari: Phytoseiidae) and implications for biological control strategies. Syst Appl Acarol.

[CR83] Mercier J, Manker DC (2005). Biocontrol of soil-borne diseases and plant growth enhancement in greenhouse soilless mix by the volatile-producing fungus *Muscodor albus*. Crop Prot.

[CR84] Messelink GJ (2014). Persistent and emerging pests in greenhouse crops: is there a need for new natural enemies. IOBC/WPRS Bull.

[CR85] Messelink GJ, de Kogel WJ (2005). Impact of chrysanthemum cultivar, fertilization and soil-dwelling predatory mites on *Frankliniella occidentalis*. Proc Neth Entomol Soc Meet.

[CR86] Messelink GJ, Holstein-Saj RV (2008). Improving thrips control by the soil-dwelling predatory mite *Macrocheles robustulus* (Berlese). IOBC/WPRS Bull.

[CR87] Messelink GJ, van Holstein-Saj R (2006). Potential for biological control of the bulb scale mite (Acari: Tarsonemidae) by predatory mites in amaryllis. Proc Neth Entomol Soc Meet.

[CR89] Messelink GJ, van Slooten M (2004). Effects of soil-dwelling predators and organic treatments on the cabbage root fly *Delia radicum* (Diptera: Anthomyiidae) in greenhouse radish. Proc Neth Entomol Soc Meet.

[CR90] Messelink GJ, van Wensveen W (2003). Biocontrol of *Duponcheria fovealis* (Lepidoptera: Pyralidae) with soil-dwelling predators in potted plants. Commun AgriCult Appl Biol Sci.

[CR91] Messelink GJ, van Maanen R, van Steenpaal SEF, Janssen A (2008). Biological control of thrips and whiteflies by a shared predator: two pests are better than one. Biol Control.

[CR92] Mills N, Brodeur J, Boivin G (2006). Interspecific competition among natural enemies and single versus multiple introductions in biological control. Trophic and guild interactions in biological control.

[CR93] Moens M, Perry RN, Starr JL, Perry RN, Moens M, Starr JL (2009). Meloidogyne species—a diverse group of novel and important plant parasites. Root-knot nematodes.

[CR94] Momen FM, Abdel-Khalek A (2009). Cannibalism and intraguild predation in the phytoseiid mites *Typhlodromips swirskii*, *Euseius scutalis* and *Typhlodromus athiasae* (Acari: Phytoseiidae). Acarina.

[CR95] Montserrat M, Magalhães S, Sabelis MW (2008). Patterns of exclusion in an intraguild predator-prey system depend on initial conditions. J Anim Ecol.

[CR96] Montserrat M, Magalhães S, Sabelis MW (2012). Invasion success in communities with reciprocal intraguild predation depends on the stage structure of the resident population. Oikos.

[CR97] Moreira GF, de Moraes GJ, Carrillo D, de Moraes G, Peña J (2015). The potential of free-living laelapid mites (Mesostigmata: Laelapidae) as biological control agents. Prospects for biological control of plant feeding mites and other harmful organisms. Progress in Biological Control.

[CR98] Moreira GF, de Morais MR, Busoli AC, de Moraes GJ (2015). Life cycle of *Cosmolaelaps jaboticabalensis* (Acari: Mesostigmata: Laelapidae) on *Frankliniella occidentalis* (Thysanoptera: Thripidae) and two factitious food sources. Exp Appl Acarol.

[CR99] Müller JL (2019) A study of root aphid *Aploneura lentisci* Pass. biology and root aphid-host interactions with perennial ryegrass/endophyte associations in New Zealand.PhD thesis, Massey University, Manawatū, New Zealand

[CR100] Muñoz-Cárdenas K (2017) What lies beneath? Linking litter and canopy food webs to protect ornamental crops. PhD Thesis, University of Amsterdam, the Netherlands

[CR101] Muñoz-Cárdenas K, Fuentes LS, Cantor RF (2014). Generalist red velvet mite predator (*Balaustium* sp.) performs better on a mixed diet. Exp Appl Acarol.

[CR102] Muñoz-Cárdenas K, Ersin F, Pijnakker J (2017). Supplying high-quality alternative prey in the litter increases control of an above-ground plant pest by a generalist predator. Biol Control.

[CR103] Navarro-Campos C, Wäckers FL, Pekas A (2016). Impact of factitious foods and prey on the oviposition of the predatory mites *Gaeolaelaps aculeifer* and *Stratiolaelaps scimitus* (Acari: Laelapidae). Exp Appl Acarol.

[CR104] Navarro-Campos C, Beltrà A, Calabuig A (2020). Augmentative releases of the soil predatory mite *Gaeolaelaps aculeifer* reduce fruit damage caused by an invasive thrips in Mediterranean citrus. Pest Manag Sci.

[CR105] Neher DA, Barbercheck ME (2019). Soil microarthropods and soil health: intersection of decomposition and pest suppression in agroecosystems. Insects.

[CR106] Nomikou M, Janssen A, Schraag R, Sabelis MW (2002). Phytoseiid predators as potential biological control agents for *Bemisia tabaci*. Exp Appl Acarol.

[CR107] Nomikou M, Sabelis MW, Janssen A (2010). Pollen subsidies promote whitefly control through the numerical response of predatory mites. Biocontrol.

[CR108] Northfield TD, Barton BT, Schmitz OJ (2017). A spatial theory for emergent multiple predator–prey interactions in food webs. Ecol Evol.

[CR109] Owens BE, Carlton CE (2015). "Berlese vs. Winkler”: comparison of two forest litter coleoptera extraction methods and the ECOLI (Extraction of Coleoptera in Litter) Protocol. Coleopterists Bull.

[CR110] Pasquier A, Andrieux T, Martinez-Rodiguez P, Vercken E, Ferrero M (2021). Predation capacity of soil-dwelling predatory mites on two major maize pests. Acarologia.

[CR111] Pasquier A, Monticelli LS, Moreau A, Kaltenbach B, Chabot C, Andrieux T, Ferrero M, Vercken E (2021). A promising predator-in-first strategy to control Western corn rootworm population in maize fields. Agronomy.

[CR112] Paulitz TC (1997). Biological control of root pathogens in soilless and hydroponic systems. HortScience.

[CR113] Perry RN, Moens M, Jones J, Gheysen G, Fenoll C (2011). Introduction to plant-parasitic nematodes; modes of parasitism. Genomics and molecular genetics of plant-nematode interactions.

[CR114] Pijnakker J, Ramakers P (2008). Predatory mites for biocontrol of Western Flower Thrips, *Frankliniella occidentalis* (Pergande), in cut roses. IOBC/WPRS Bull.

[CR115] Pijnakker J, Ramakers P (2009). Development of integrated pest management in greenhouse cut roses (in the Netherlands). Floricult Ornam Biotechnol.

[CR116] Pijnakker J, Arijs Y, de Souza A (2016). The use of *Typha angustifolia* (Cattail) pollen to establish the predatory mites *Amblyseius swirskii*, *Iphiseius degenerans*, *Euseius ovalis* and *Euseius gallicus* in glasshouse crops. IOBC/WPRS Bull.

[CR117] Pijnakker J, Leman A, Vangansbeke D, Wackers F (2017). *Echinothrips americanus*: a bottleneck for integrated pest management in ornamentals?. Commun Agric Appl Biol Sci Ghent.

[CR118] Pochubay E, Tourtois J, Himmelein J, Grieshop M (2015). Slow-release sachets of *Neoseiulus cucumeris* predatory mites reduce intraguild predation by *Dalotia coriaria* in greenhouse biological control systems. Insects.

[CR119] Pozzebon A, Boaria A, Duso C (2015). Single and combined releases of biological control agents against canopy- and soil-dwelling stages of *Frankliniella occidentalis* in cyclamen. Biocontrol.

[CR120] Premachandra WTSD, Borgemeister C, Berndt O (2003). Combined releases of entomopathogenic nematodes and the predatory mite *Hypoaspis aculeifer* to control soil-dwelling stages of western flower thrips *Frankliniella occidentalis*. Biocontrol.

[CR121] Prischmann-Voldseth DA, Dashiell KE (2013). Effects of releasing a generalist predator (Acari: *Gaeolaelaps aculeifer*) on a subterranean insect herbivore (Coleoptera: *Diabrotica virgifera virgifera*). Biol Control.

[CR122] Prischmann-Voldseth DA, Knutson EM, Dashiell KE, Lundgren JG (2011). Generalist-feeding subterranean mites as potential biological control agents of immature corn rootworms. Exp Appl Acarol.

[CR123] Rahman T, Spafford H, Broughton S (2012). Use of spinosad and predatory mites for the management of *Frankliniella occidentalis* in low tunnel-grown strawberry. Entomol Exp Appl.

[CR124] Ramakers PJM (1980). Biological control of *Thrips tabaci* (Thysanoptera: Thripidae) with *Amblyseius* spp. (Acari: Phytoseiidae). Bull SROP/WPRS.

[CR125] Ramakers PJM, van Lieburg MJ (1982). Start of commercial production and introduction of *Amblyseius mckenziei* Sch. and Pr. (Acarina: Phytoseiidae) for the control of *Thrips tabaci* Lind. (Thysanoptera: Thripidae) in glasshouses. Mededelingen Faculteit Landbouwwetenschappen Rijksuniversiteit Gent.

[CR126] Rasmussen JJ, Nørum U, Jerris MR (2013). Pesticide impacts on predator-prey interactions across two levels of organisation. Aquat Toxicol.

[CR127] Relyea RA, Edwards K (2010). What doesn’t kill you makes you sluggish: how sublethal pesticides alter predator–prey interactions. Copeia.

[CR128] Riedell WE (1990). Rootworm and mechanical damage effects on root morphology and water relations in maize. Crop Sci.

[CR129] Riley DG, Joseph SV, Srinivasan R, Diffie S (2011). Thrips vectors of Tospoviruses. J Integr Pest Manage.

[CR130] Roberts JMK, Weeks AR, Hoffmann AA, Umina PA (2011). Does *Bdellodes lapidaria* (Acari: Bdellidae) have a role in biological control of the springtail pest, *Sminthurus viridis* (Collembola: Sminthuridae) in south-eastern Australia?. Biol Control.

[CR131] Rosenheim JA, Kaya HK, Ehler LE (1995). Intraguild predation among biological-control agents: theory and evidence. Biol Control.

[CR132] Rueda-Ramírez D, Rios-Malaver D, Varela-Ramírez A, Moraes GJ (2018). Colombian population of the mite *Gaeolaelaps aculeifer* as a predator of the thrips *Frankliniella occidentalis* and the possible use of an astigmatid mite as its factitious prey. Syst Appl Acarol.

[CR133] Rueda-Ramírez D, Rios‐Malaver D, Varela‐Ramírez A, Moraes GJ (2019). Biology and predation capacity of *Parasitus bituberosus* (Acari: Mesostigmata: Parasitidae) on *Frankliniella occidentalis* (Thysanoptera: Thripidae), and free‐living nematodes as its complementary prey. Pest Manag Sci.

[CR134] Sabelis MW, van Rijn PCJ, Lewis T (1997). Predation by insects and mites. Thrips as crop pests.

[CR135] Sabelis MW, Lesna I, Aratchige NS (2007). A tritrophic perspective to the biological control of Eriophyoid mites. IOBC/WPRS Bull.

[CR136] Sabelis MW, Janssen A, Lesna I (2008). Developments in the use of predatory mites for biological pest control. IOBC/WPRS Bull.

[CR137] Sabu TK, Shiju RT, Vinod K, Nithya S (2011). A comparison of the pitfall trap, Winkler extractor and Berlese funnel for sampling ground-dwelling arthropods in tropical montane cloud forests. J Insect Sci.

[CR138] Saito T, Brownbridge M (2016). Compatibility of soil-dwelling predators and microbial agents and their efficacy in controlling soil-dwelling stages of western flower thrips *Frankliniella occidentalis*. Biol Control.

[CR139] Scherber C, Eisenhauer N, Weisser WW (2010). Bottom-up effects of plant diversity on multitrophic interactions in a biodiversity experiment. Nature.

[CR140] Scheu S (2001). Plants and generalist predators as links between the below-ground and above-ground system. Basic Appl Ecol.

[CR141] Sikora RA, Coyne D, Hallman J, Timper P, Sikora RA, Coyne D, Hallman J, Timper P (2018). Reflections and challenges: nematology in subtropical and tropical agriculture. Plant parasitic nematodes in subtropical and tropical agriculture.

[CR142] Soares MA, Campos MR, Passos LC (2019). Botanical insecticide and natural enemies: a potential combination for pest management against *Tuta absoluta*. J Pest Sci.

[CR143] Soroka JJ, Kuhlmann U, Floate KD, Mason PG, Huber JT (2001). *Delia radicum* (L.), Cabbage Maggot (Diptera: Anthomyiidae). Biological control programmes in Canada.

[CR144] Spencer JL, Hibbard BE, Moeser J, Onstad DW (2009). Behaviour and ecology of the western corn rootworm (*Diabrotica virgifera virgifera* LeConte). Agric For Entomol.

[CR145] Spike BP, Tollefson JJ (1991). Yield response of corn subjected to western corn root worm (Coleoptera: Chrysomelidae) infestation and lodging. J Econ Entomol.

[CR146] Srinivasan R (2012). Integrating biopesticides in pest management strategies for tropical vegetable production. J Biopesticides.

[CR147] Steiner MY, Spohr LJ, Goodwin S (2011). Relative humidity controls pupation success and dropping behaviour of western flower thrips, *Frankliniella occidentalis* (Pergande) (Thysanoptera: Thripidae). Aust J Entomol.

[CR148] Stocks SD, Hodges A (2012) European pepper moth or southern European marsh *Pyralid Duponchelia fovealis* (Zeller). Department of Entomology and Nematology; UF/IFAS Extension. Gainesville, FL 32611 Document EENY-508 10

[CR149] Sutter GR, Fisher JR, Elliott NC, Branson TF (1990). Effect of insecticide treatments on root lodging and yields of maize in controlled infestations of western corn rootworms (Coleoptera: Chrysomelidae). J Econ Entomol.

[CR150] Thoeming G, Poehling HM (2006). Integrating soil-applied azadirachtin with *Amblyseius cucumeris* (Acari: Phytoseiidae) and *Hypoaspis aculeifer* (Acari: Laelapidae) for the management of *Frankliniella occidentalis* (Thysanoptera: Thripidae). Environ Entomol.

[CR151] Tommasini MG, Maini S (1995). *Frankliniella occidentalis* and other thrips harmful to vegetable and ornamental crops in Europe. Wagening Agric Univ Papers.

[CR152] Ullman DE, Meideros R, Campbell LR (2002). Thrips as vectors of tospoviruses. Adv Bot Res.

[CR153] van Lenteren JC (2012). The state of commercial augmentative biological control: plenty of natural enemies, but a frustrating lack of uptake. Biocontrol.

[CR154] van Maanen R, Messelink GJ, Van Holstein-Saj R (2012). Prey temporarily escape from predation in the presence of a second prey species. Ecol Entomol.

[CR155] van Rijn PC, Van Houten YM, Sabelis MW (1999). Pollen improves thrips control with predatory mites. IOBC/WPRS Bull.

[CR156] van Rijn PCJ, van Houten YM, Sabelis MW (2002). How plants benefit from providing food to predators even when it is also edible to herbivores. Ecology.

[CR157] van Schelt J, Mulder S (2000). Improved methods of testing and release of *Aphidoletes aphidimyza* (Diptera: Cecidomyiidae) for aphid control in glasshouses. Eur J Entomol.

[CR158] van Schelt J, Hoogerbrugge H, van Houten Y, Blockmans K (2002). Biological control and survival of *Echinothrips americanus* in pepper. IOBC/WPRS Bull.

[CR159] Vance-Chalcraft HD, Rosenheim JA, Vonesh JR (2007). The influence of intraguild predation on prey suppression and prey release: a meta-analysis. Ecology.

[CR160] Velthuis HHW, Van Doorn A (2006). A century of advances in bumblebee domestication and the economic and environmental aspects of its commercialization for pollination. Apidologie.

[CR161] von Berg K, Thies C, Tscharntke T, Scheu S (2009). Cereal aphid control by generalist predators in presence of belowground alternative prey: complementary predation as affected by prey density. Pedobiologia.

[CR162] Waiganjo MM, Waturu CN, Mureithi JM (2011). Use of entomopathogenic fungi and neem bio-pesticides for brassica pests control and conservation of their natural enemies. East Afr Agric For J.

[CR163] Walter DE, Stirling GR (2018). Microarthropods in Australian sugarcane soils: a survey with emphasis on the mesostigmata as potential regulators of nematode populations. Acarologia.

[CR164] Warburg S, Inbar M, Gal S (2019). The effects of a windborne pollen-provisioning cover crop on the phytoseiid community in citrus orchards in Israel. Pest Manag Sci.

[CR165] Wardle DA, Bardgett RD, Klironomos JN, Setälä H, van der Putten WH, Wall DH (2004). Ecological linkages between aboveground and belowground biota. Science.

[CR166] Wenninger EJ (2011) Sugar beet root aphids: identification, biology, & management. University of Idaho Extension, University of Idaho. CIS 1176

[CR167] Wiethoff J, Poehling HM, Meyhöfer R (2004). Combining plant- and soil-dwelling predatory mites to optimise biological control of thrips. Exp Appl Acarol.

[CR168] Woltz JM, Isaacs R, Landis DA (2012). Landscape structure and habitat management differentially influence insect natural enemies in an agricultural landscape. Agric Ecosyst Environ.

[CR169] Wright EM, Chambers RJ (1994). The biology of the predatory mite *Hypoaspis miles* (Acari: Laelapidae), a potential biological control agent of *Bradysia paupera* (Dipt. Sciaridae) Entomophaga.

[CR170] Wu S, Gao Y, Xu X, Wang E, Wang Y, Lei Z (2014). Evaluation of *Stratiolaelaos scimitus* and *Neoseiulus barkeri* for biological control of thrips on greenhouse cucumbers. Biocontrol Sci Technol.

[CR171] Wu S, Zhang Z, Gao Y (2016). Interactions between foliage- and soil-dwelling predatory mites and consequences for biological control of *Frankliniella occidentalis*. Biocontrol.

[CR172] Wu S, Xing Z, Ma T (2021). Competitive interaction between *Frankliniella occidentalis* and locally present thrips species: a global review. J Pest Sci.

[CR173] Xu CL, Chen YL, Xu XN (2014). Evaluation of *Blattisocius dolichus* (Acari: Blattisociidae) for biocontrol of root-knot nematode, *Meloidogyne incognita* (Tylenchida: Heteroderidae). Biocontrol.

[CR174] Yan H, Zhang B, Wang E, Xu X, Wei GS (2022). Combining predatory mites and film mulching to control *Bradysia cellarum* (Diptera: Sciaridae) on Chinese chives, *Allium tuberosum*. Exp Appl Acarol.

[CR175] Yang S-H, Wang D, Chen C (2020). Evaluation of *Stratiolaelaps scimitus* (Acari: Laelapidae) for controlling the root-knot nematode, *Meloidogyne incognita* (Tylenchida: Heteroderidae). Sci Rep.

[CR176] Zhang X, Wu S, Reitz SR, Gao Y (2021). Simultaneous application of entomopathogenic *Beauveria bassiana* granules and predatory mites *Stratiolaelaps scimitus* for control of western flower thrips, *Frankliniella occidentalis*. J Pest Sci.

